# Oral cancer diagnosis based on gated recurrent unit networks optimized by an improved version of Northern Goshawk optimization algorithm

**DOI:** 10.1016/j.heliyon.2024.e32077

**Published:** 2024-05-29

**Authors:** Lei Zhang, Rongji Shi, Naser Youssefi

**Affiliations:** a Department of Stomatology, The Second Hospital, Cheeloo College of Medicine, Shandong University, Jinan, 250033, Shandong, China; b Islamic Azad University, Science and Research Branch, Tehran, Iran; c College of Technical Engineering, The Islamic University, Najaf, Iraq

**Keywords:** Oral cancer, Diagnosis, Gated recurrent unit networks, Northern goshawk optimization algorithm, Recurrent neural networks, Medical imaging, Deep learning, Machine learning, Artificial intelligence

## Abstract

Oral cancer early diagnosis is a critical task in the field of medical science, and one of the most necessary things is to develop sound and effective strategies for early detection. The current research investigates a new strategy to diagnose an oral cancer based upon combination of effective learning and medical imaging. The current research investigates a new strategy to diagnose an oral cancer using Gated Recurrent Unit (GRU) networks optimized by an improved model of the NGO (Northern Goshawk Optimization) algorithm. The proposed approach has several advantages over existing methods, including its ability to analyze large and complex datasets, its high accuracy, as well as its capacity to detect oral cancer at the very beginning stage. The improved NGO algorithm is utilized to improve the GRU network that helps to improve the performance of the network and increase the accuracy of the diagnosis. The paper describes the proposed approach and evaluates its performance using a dataset of oral cancer patients. The findings of the study demonstrate the efficiency of the suggested approach in accurately diagnosing oral cancer.

## 1 Introduction

### 1.1 Oral cancer: prevalence and diagnosis

Oral cancer is a kind of cancer which grows in throat or mouth tissues, which can be called cancer of oral cavity or mouth, and can affect areas such as the gums, floor or roof of the mouth, cheeks, tongue, lips, throat, and tonsils [1]. Early diagnosis and cure, which has been considered crucial as cancer of oral, can be life-threatening [2]. Medical imaging techniques such as MRI (Magnetic Resonance Imaging), PET (Positron Emission Tomography), and CT (Computed Tomography) are vital to diagnose mouth cancer at the beginning stages [3].

CT scans while making use of X-rays give detailed images of the oral cavity, including its blood vessels (arteries), soft tissues, and bones. The existence of oral cancer can be determined by CT scans by looking for abnormal growths or tumors in the mouth, throat, or neck [4]. MRI scans utilize radio weaves and powerful magnetic fields to present some images, which are of the mouth cavity that reveal more specific details about the soft tissues [5]. Throat or mouth tumors can be found with an MRI scan, along with any changes in the surrounding tissue that can point to the presence of malignancy [6].

PET scans track the body's metabolic activities by using a little quantity of radioactive materials. Because cancer cells have greater metabolic activity than healthy cells, PET scans can locate potential cancerous regions in the body even before a tumor develops [7]. To confirm the presence of malignant cells in the oral cavity, clinicians can carry out a biopsy in addition to these imaging modalities. There is a practice called biopsy in which a tiny tissue sample is taken from the afflicted area and is inspected under a microscope [8]. However, these methods can be time-consuming and invasive that can have restrictions with regard to effectiveness and the precision. Therefore, oral cancer diagnosis requires efficient and meticulous strategies [9].

### 1.2 Literature review

Recent developments in artificial Intelligence and machine learning demonstrate a promise for increasing the precision and effectiveness of cancer detection [10]. For instance, the objective of Ilhan et al. [11] in 2020 was to assess the potential benefits of creating optical imaging techniques and some other strategies, which were based on AI for detecting mouth cancer and enhancing consequences. The paper gave a summary of clinical requirements and parameters of image-based approaches for identifying oral cancer. It also included a concise overview of research that employed these techniques separately or in combination, as well as information on artificial intelligence systems. While some cutting-edge imaging techniques have been researched to improve oral cancer outcomes, none had yet been widely adopted or had a major influence on practice or results. The study showed that merging artificial intelligence with imaging might significantly increase diagnostic accuracy, despite a lack of studies that focused on oral cancer.

Recently, there were several works in terms of using the AI methods for this purpose [12]. Moreover, combining imaging and AI techniques might increase diagnosis precision, providing patients with oral cancer with improved options for therapy.

Warin et al. [13] in 2021 developed an automatic classification model to detect mouth cancer screening utilizing CNN (Convolutional Neural Network) that advanced learning algorithms. 700 oral photographs were used, 350 of which were oral squamous cell carcinoma and the rest were normal mouth mucosa. In order to classify and detect the illnesses, DenseNet21 and R–CNN were, in turn, utilized. The classification precision achieved a precision, recall, F1-score, sensitivity, specificity, and AUC of 99 %, 100 %, 99 %, 98.75 %, 100 %, and 99 %, respectively. The accuracy of detection achieved a precision, recall, F1-score, and AUC of 76.67 %, 82.14 %, 79.31 %, and 0.79 respectively.

In 2022, Panigrahi et al. [14] suggested a different method for the classification of mouth cancer using the capsule network, a deep learning technique. The capsule network was strengthened for rotation and affine transformation of the expanded oral information by dynamic routing and routing by agreement. The network's ability to handle orientation, position, and outlook made it appropriate for the early-stage analysis of mouth cancer images which were histopathological. The cross-validation performance indicated that the suggested strategy successfully classified the histopathological images of OSCC (Oral Squamous Cell Carcinoma) with sensitivity, specificity, and accuracy of 97.78 %, 96.92 %, and 97.35 %, respectively.

In 2023, Haron et al. [15] assessed the effectiveness of a phone application called MeMoSA® to identify possible mouth cancers and malignant disorders. A prospective research was carried out with 355 participants; 280 of them were diagnosed with oral variants or lesions. MeMoSA® was contrasted to another oral test using kappa statistics based on some factors, like sensitivity, intra-rater agreement, concordance, F1-score, and specificity. The study found that specificity and sensitivity of MeMoSA® were, in turn, 95.5 % and 94.0 %, for referral decisions with inter-rater agreement of 0.825. An off-site expert reviewed the images; moreover, MeMoSA® documented regression or progression of the lesions systematically.

In 2023, Gomes et al. [16] aimed to develop a CNN model to arrange six categories of oral images of lesion automatically. Four architectures were tested, and the InceptionV3 achieved the best results, whose correct predictions and average accuracy were more than 71 % and 95.09 %, respectively. The CNN model demonstrated satisfactory performance, which has potential for future use in identifying potentially malignant, malignant, and benign oral lesions.

As can be observed from the literature, different models based on AI are proposed to the diagnose the oral cancer. Table 1 illustrates the key features of the literature.

**Table 1:** The key features of the literature.

Study	Key Features
Ilhan et al. (2020)	Raman spectroscopy and autofluorescence imaging techniques were utilized. Both methods were combined for improving sensitivity and specificity.
Warin et al. (2021)	CNN-based models (DenseNet121 and ResNet-50) were combined for categorizing Oral Potentially Malignant Disorders (OPMDs) with high sensitivity and specificity.
Panigrahi et al. (2022)	A capsule network was proposed for oral squamous cell carcinoma classification, which was robust to rotation and affine transformations.
Haron et al. (2023)	The MeMoSA® mobile app was introduced for oral cancer identification using image review with high referral accuracy for early management.
Gomes et al. (2023)	Vision Transformer (ViT) and Swin Transformer were employed for mobile-based oral cancer image classification that could Achieve high accuracy rates.

The present research showcases progress in artificial intelligence methods for identifying, categorizing, and forecasting oral cancer, which aids in prompt treatment and better results for patients.

A new approach was explored for detecting oral cancer through the utilization of Gated Recurrent Unit (GRU) networks that have been fine-tuned by an enhanced version of the Northern Goshawk Optimization (NGO) algorithm. The benefits of this method encompass the ability to analyze extensive and intricate datasets, achieve high levels of accuracy, and identify cancer at an early stage. The upgraded NGO algorithm plays a crucial role in boosting the performance of GRU networks and improving diagnostic precision.

### 1.3 Motivation

One of the most widely-used and effective artificial intelligence tools is the Artificial Neural Network (ANN). Among them, Deep Neural Networks (DNN) have been observed to provide the highest efficiency and satisfactory results for various applications, including image and speech recognition. Sequential data can be processed by a kind of neural network called RNNs (Recurrent Neural Networks); moreover, RNNs have demonstrated promising findings in the medical field to diagnose and predict illness. Gated Recurrent Unit (GRU) networks, a kind of Recurrent neural network, can manage dependencies which are long-term and have also been used in the medical field for various applications.

A metaheuristic optimization algorithm is a promising strategy for addressing optimization problems. One such algorithm is the Northern Goshawk Optimization (NGO) algorithm, which draws inspiration from the foraging behavior of Northern Goshawks. The NGO algorithm has demonstrated successful outcomes in various optimization problems and has shown potential in enhancing the performance of machine learning algorithms.

Recently, numerous investigations have been conducted on the use of neural networks optimized by metaheuristic algorithms to diagnose oral cancer. These studies have demonstrated that the combination of networks and algorithms can enhance the precision and effectiveness to diagnose oral cancer. Nonetheless, myriad investigations are necessary to assess the potential of the current approach in clinical settings to distinguish its performance with other modern methods. The goal of the current essay is to propose a novel approach to diagnose oral cancer by utilizing GRU networks optimized by an enhanced version of the NGO algorithm. The suggested strategy intends to advance the precision and effectiveness of oral cancer diagnosis while also offering a non-intrusive and economical approach to diagnose oral cancer.

### 1.4 Contribution

Oral cancer is a significant public health issue worldwide, and if patients are timely detected and diagnosed, the chances of successful cure enhances. The utilization of ML (Machine Learning) with AI techniques, such as GRU networks optimized by an improved model of NGO algorithm, has demonstrated promise to enhance the precision and effectiveness of diagnosing oral cancer. The proposed approach in this research paper aims to provide a non-intrusive and economical strategy for diagnosing oral cancer; moreover, it has demonstrated high accuracy and specificity in diagnosing oral cancer. Further research can explore the clinical implementation of the proposed approach and validate its effectiveness in larger datasets.

The paper provides a detailed description of the suggested method and the methodology used to evaluate its efficiency. The dataset used in the study consists of oral cancer patients, and the findings illustrate the efficiency of the suggested method in accurately diagnosing oral cancer. The study also highlights the potential of the proposed approach to improve the overall detection and treatment of oral cancer. The timely detection of oral cancer can significantly enhance the chances of successful treatment, and the proposed approach can help achieve this goal.

## 2 Dataset description

In this research study, the “Oral Cancer (Lips and Tongue) images” information was utilized to analyze and evaluate the proposed method. The dataset consists of captured images from two distinct groups, comprising cancerous and non-cancerous, which can be employed to validate various types of diagnosis systems. The dataset comprises 87 sets of oral cancer images representing the cancerous group and 44 sets of oral non-cancer images representing the non-cancerous group. These images were captured by several Ear, Nose, and Throat (ENT) hospitals in Ahmedabad. To ensure accurate classification, the images were carefully examined and classified by ENT doctors, who provided their expertise in determining the presence or absence of oral cancer.

All images in the dataset are saved in the widely-used “JPEG” format, denoted by the extension “*.jpg”. Each image represents a specific case and offers visual information related to the presence or absence of oral cancer. The dataset provides a valuable resource for researchers, clinicians, and developers seeking to investigate and develop diagnostic systems of oral cancer.

Access to the dataset is available through the Kaggle website, where it is hosted [17]. Researchers can retrieve the dataset from the specified URL and utilize it for various purposes, such as training machine learning models, developing computer-aided diagnosis systems, and conducting further studies in the context of detecting oral cancer.

The “Oral Cancer (Lips and Tongue) images” dataset offers a valuable contribution to the research community and provides a foundation for advancing the understanding of oral cancer diagnosis and treatment. By using this dataset, researchers can explore novel approaches, algorithms, and techniques to upgrade the meticulousness and efficiency in detection of oral cancer, potentially causing timely diagnoses and enhanced patient consequences.

**Fig. 1:**
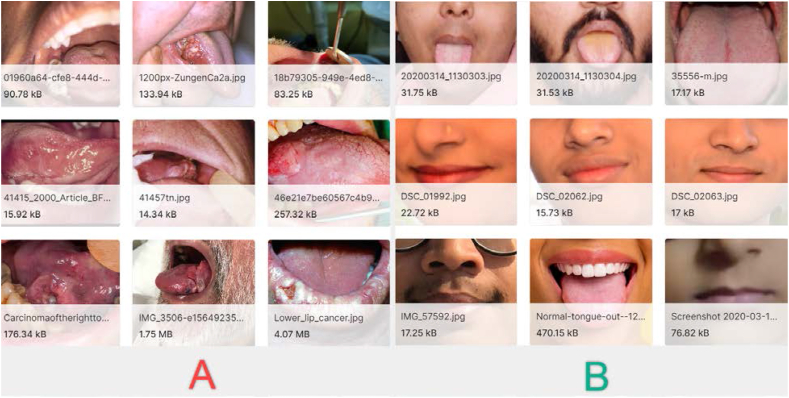
The selection of sample images from the Oral Cancer (OCI) dataset, encompassing both (A) cancerous and (B) non-cancerous cases [17].

Fig. (1(A, B)) displays the selection of sample images from the Oral Cancer Images (OCI) dataset, encompassing both cancerous and non-cancerous cases [17].

To confirm the diagnosis of oral cancer, a thorough physical examination of the patient's oral cavity is first conducted. Diagnosing oral cancer involves a combination of clinical examination, imaging tests, and tissue biopsy. While there is no official screening test for oral cancer, periodic oral self-exams are recommended to look for suspicious signs, such as abnormal lumps, sores, or color changes in the lips, gums, cheeks, and tongue. If anything is detected, an appointment should be scheduled promptly with an Ear-Nose-Throat (ENT) doctor. During routine healthcare visits, an ENT doctor, family physician, or dentist performs a head and neck examination, using a light and mouth mirror to visualize all surfaces inside the mouth and assess any abnormalities, such as sores, white patches (leukoplakia), or other changes. Here, the data has been separated into two folders, including “cancerous” and “non-cancerous”.

## 3 Methodology

In this study, a method for oral cancer recognition is proposed based on JPEG. The methodology consists of four main stages, namely preprocessing, feeding the preprocessed data to the Gated Recurrent Unit (GRU) network, optimizing the network using the Northern Goshawk Optimization with Lévy flight and Chaos theory (NGO-LC), and final classification.

### 3.1 Preprocessing

In the initial stage of data preprocessing, images in JPEG format of oral cancer related to lip and tongue are imperiled to three techniques. These techniques include Random Over Sampling (ROS), data augmentation, and image resizing. The ROS technique is used to conduct the unbalanced data sets problem, where positive cases (oral cancer) meaningfully outnumber negative cases (non-cancer). Some techniques under the exclusive of data augmentation are employed to rise the size of a dataset unnaturally by performing a variety of transformations to the existing data. Moreover, an image resizing technique is used to ensure the standardization of image dimensions before entering the Gated Recurrent Unit network.

### 3.2 Feeding the preprocessed data to the GRU network

The preprocessed data is subsequently used to train the GRU network. The GRU network is a kind of RNN that is particularly built to process sequential input, making it appropriate for processing oral images. The network undergoes training using preprocessed JPEG images to distinguish between malignant and non-cancerous instances.

### 3.3 Optimizing the network using NGO-LC

The Northern Goshawk Optimization method with Lévy flight and chaos theory is utilized to optimize the framework of the Gated Recurrent Unit network. The hunting habit of the Northern Goshawk, a superb bird of prey recognized for its quickness and agility in flight, inspired this algorithm. To achieve a balance between exploration and making use of the solution space, the algorithm incorporates the benefits of both chaos theory and Lévy flight. This lets the GRU network discover the ideal structure based on its weight, improving its efficacy in oral cancer identification.

### 3.4 Final classification

The Gated Recurrent Unit network is used for the final categorization of the oral pictures after it has been optimized using NGO-LC. It uses the learnt characteristics and weights to categorize the photos as malignant or non-cancerous. The study's suggested technique attempts to enhance oral cancer detection utilizing JPEG scans of the lips and tongue. The combination of preprocessing approaches, the GRU network and the NGO-LC optimization algorithm, is intended to improve classification accuracy and efficacy. Here, the pseudocode the suggested JPEG-based technique for the oral cancer identification has been illustrated using a GRU network with NGO-LC method:

**Algorithm 1:** Oral Cancer Recognition

Input: Oral cancer images in JPEG format
1. Preprocessing:
1.1. Apply Random Over Sampling (ROS) to address imbalanced datasets.
1.2. Perform techniques of data augmentation to enhance size of the dataset.
1.3. Resize the images to a standardized dimension.
2. Feed Preprocessed Data to GRU Network:
2.1. Train a Gated Recurrent Unit (GRU) network on the preprocessed data.
2.2. Input the preprocessed JPEG images into the GRU network.
2.3. Train the GRU network to distinguish between cancerous and non-cancerous cases.
3. Optimize the Network Using NGO-LC:
3.1. Apply Northern Goshawk Optimization with Lévy flight and Chaos theory (NGO-LC).
3.2. Use the NGO-LC algorithm to optimize the structure of the GRU network based on its weights.
4. Final Classification:
4.1. Utilize the optimized GRU network for the final classification.
4.2. Classify the oral images as malignant or non-cancerous based on the learned features and weights.
Output: Classification results indicating whether the oral images are malignant or non-cancerous

In the following, the different parts of this process have been explained.

## 4 Preprocessing

Image preprocessing methods, such as random oversampling (ROS), data augmentation, and image scaling, are critical in enhancing the efficacy of systems for diagnosing oral cancer images. Here a breakdown of each strategy has been revealed and how it helps improve system performance:

### 4.1 ROS (Random Over Sampling)

By recognizing this fact that the dataset suffers from class imbalance, where the number of cancerous image cases is 87, while the number of non-cancerous image cases is 44. Class imbalance can lead to biased model training and lower performance in detecting the minority class, which in this case is the cancerous images.

ROS is a method employed to handle imbalanced data sets in which the amount of positive cases (oral cancer) is much lower than the number of negative instances (non-cancerous). Imbalanced datasets might result in a biased model training and poor detection of the minority class. To balance the dataset, Random Over Sampling requires replicating instances from the minority class. By increasing the number of positive instances, the model obtains enough training on the oral cancer pictures, allowing for improved accuracy in classification and lowering the probability of misclassifying malignant cases as non-cancerous.

Therefore, in this study, duplicating some of the non-cancerous images to increase their representation in the dataset is required. The oversampled non-cancerous images have been randomly selected from the original dataset until the number of cancerous and non-cancerous samples duplicate them so that they have become the same. Here, the number of cancerous image cases is 87, therefore, we need to add 43 (87–44) oversampling non-cancerous image cases to provide the same number of cases.

**Fig. 2:**
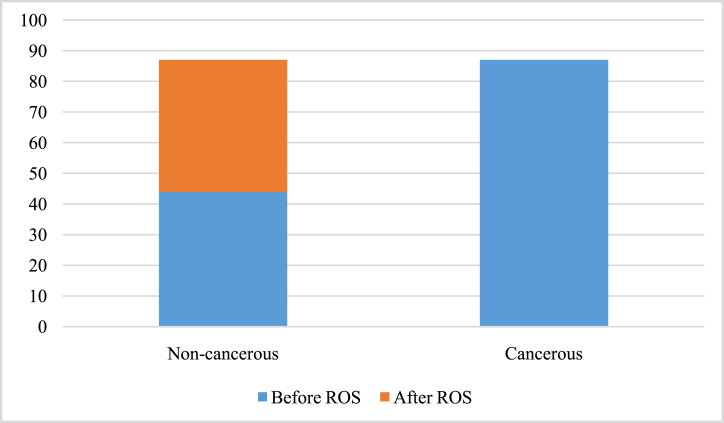
Distribution of classes in a dataset prior to and subsequent to using an over-sampling strategy.

The results in Fig. (2) demonstrate that how classes are distributed in a dataset prior to and subsequent to using an over-sampling strategy. The classifications under consideration include “non-cancerous” and “cancerous” images, which most likely correspond to either the existence or lack of oral cancer in JPEG photos.

### 4.2 Data augmentation

Data augmentation is applying different randomized alterations to existing data to artificially expand the dataset size. Augmentation of data helps the model generalize better to different variants and decreases the danger of being overfit by incorporating these variances. Data augmentation can imitate differences in image acquisition settings, such as varied lighting conditions, angles, or orientations of the mouth pictures, in the context of oral cancer imaging diagnosis. This allows the model to learn more robust characteristics that are more reflective of real-world settings, resulting in better performance in categorizing oral cancer images. In this study, Resizing, Warping, Cropping, Simulation noise, Simulation blur, Jitter color, and Translation have been used for images augmentation, which are explained in details in the following.

#### 4.2.1 Resizing

Resizing involves adjusting the dimensions of an image while maintaining its aspect of ratio. It can be useful for standardizing the size of images in a dataset. In the present research, resizing has been likely applied to ensure that all oral cancer images have a consistent dimension before further processing. In this study, [0.5, 2, 3] resizing times are used for augmentation.

#### 4.2.2 Warping

Warping refers to geometric transformations that distort the shape of an image. The warping was applied between the range [40,60] degrees, possibly varying the amount of distortion. Warping can help introduce variations in images of oral cancer, making the model more robust to different shapes and deformations.

#### 4.2.3 Cropping

Cropping involves selecting a specific region of interest from an image by removing unwanted portions. It helps focus the model's attention on the relevant features of the oral cancer images and discard irrelevant or distracting background information.

#### 4.2.4 Simulation noise

Adding simulated noise to images introduces random variations in pixel values. Noise can simulate real-world imperfections in image acquisition and enhance the model's ability to be generalized to noisy or less pristine images. The level of noise added in this research is based on σ=0.2, with a higher value indicating more pronounced noise.

#### 4.2.5 Simulation blur

Simulation blur involves introducing a level of blurriness to images. In this study, Gaussian blur with σ=6 is utilized. This technique can mimic out-of-focus images, which can occur in real-world scenarios. By training the model on blurred images, it can become more robust to variations in image clarity and improve its generalization capabilities.

#### 4.2.6 Jitter color

Jittering color involves modifying the color attributes of the images. Here, some modifications have been conducted, namely a Contrast to 0.4, Hue to 0.1, Saturation to 0.2, and Brightness to 0.3. This technique helps the model learn invariant features across different color variations, making it more adaptable to changes in lighting conditions or color distributions.

#### 4.2.7 Translation

Translation refers to shifting an image along the horizontal or vertical axis. By randomly translating images within a specified range, the model can learn to recognize oral cancer features regardless of their position within the image. In this study, random transition between the range [−50,50] pixels has been assumed. By applying these data augmentation techniques, the aim was to enhance the performance of the oral cancer detection model.

**Fig. 3:**
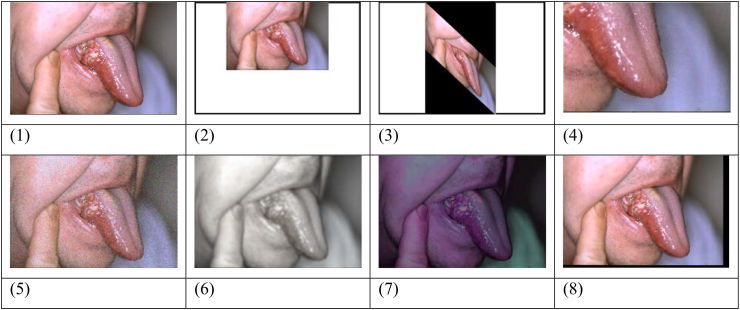
Instances of original images that have undergone data augmentation techniques: (1) is original image, and (2) to (8) are augmented images.

These techniques introduce variations, deformations, and imperfections to the images, allowing the model to learn from a more diverse and representative dataset. The purpose is to enhance the model's capability to be generalized; moreover, the aim was to accurately classify oral cancer images, even in the presence of variations in shape, noise, blur, color, and spatial positioning. It is important to note that the specific parameter values mentioned (e.g., sigma values) may vary depending on the research implementation and dataset characteristics. Fig. (3) depicts examples of original images that have undergone data augmentation techniques.

Fig. (3) presents a series of examples that illustrate how data augmentation techniques can be used to create new images from an original image. The original image is shown in (A), while the augmented images are shown in (B–H). These augmented images are produced by applying a variety of transformations to the original image, including Resizing, Warping, Cropping, Simulation noise, Simulation blur, Jitter color, and Translation. By using data augmentation techniques, variety and size of a training dataset may increase, which can help machine learning models be generalized, and it helps the models perform more effectively. The examples shown in Fig. (3) demonstrate how small changes to the original image can produce a variety of new images that capture different aspects of the underlying data.

### 4.3 Image resizing

Image resizing is the process of resizing images to a defined size. Resizing guarantees that all input photos have the same dimensions before feeding them into the model in the context of oral cancer image diagnosis. Standardizing picture sizes can assist to address challenges caused by differences in image resolutions and aspect ratios. Resized images provide the model with consistent inputs, helping it learn features more efficiently and minimizing the computational cost. Furthermore, scaling can aid in the reduction of noise or extraneous information in photos, concentrating the model's attention on the important visual patterns linked with oral cancer.

The efficacy of the system for oral cancer image diagnosis can be greatly enhanced by adding these image preprocessing techniques. Data augmentation improves the model's capacity to be generalized to diverse variants, while image scaling assures consistent inputs. By enhancing the model's capacity to extract significant characteristics from pictures and tolerate fluctuations in image collecting settings, these strategies lead to a more robust and efficient oral cancer diagnostic system.

## 5 Gated recurrent unit networks

GRU networks are a kind of RNN that Cho et al. [18] presented in 2014 as a less complex variable to LSTM (Long Short-Term Memory) networks. GRUs, like LSTMs, are intended to overcome the disappearing problem in recurrent neural netwoks, which can make learning long-term dependencies problematic.

**Fig. 4:**
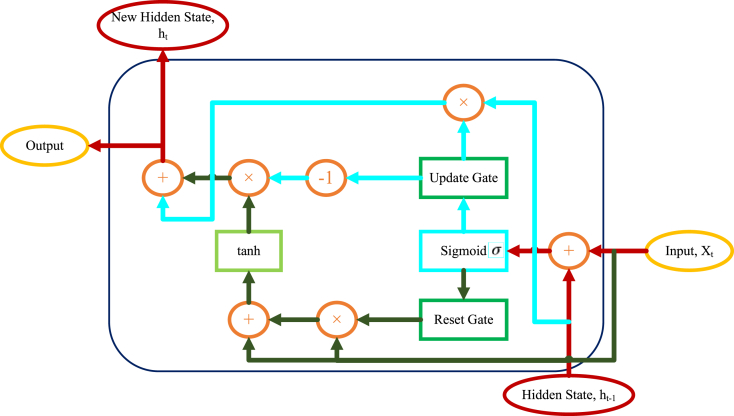
Main structure of the GRU cell.

The gates of input, update, and reset are the three basic parts that make up a GRU's structure [19]. The amount of the novel input that ought to be included in the present concealed status will be ascertained by the gate of input [20]. The gate of update is in charge of choosing what proportion of the prior concealed status should be kept and what proportion of the novel input ought to be merged into the concealed status which exist. The proportion of the former concealed status that should be forgotten is determined by the gate of reset. The main design of the cell of GRU is illustrated in Fig (4).

### 5.1 Input gate

In a GRU, the gate of input ascertains the proportion of the new input that ought to be included in the existing concealed status. The input gate receives input which exists now. Moreover, the former concealed status as outputs and inputs ranging from 0 to 1 illustrates the percentage of the novel input that should incorporate into the current hidden state. The number 0 illustrates that no novel data are added to the current concealed status, whereas the value 1 illustrates that all novel data are included in the current hidden state. Mathematically, the input gate can be defined as follows [equation (1)]:

(1) $$i_{t}=sigmoid\left(W_{i}\;\left[h_{t-1},x_{t}\right]+b_{i}\right)$$

where, $h_{t-1}$ describes the former concealed status, $x_{t}$ is the input which exists now, $W_{i}$ and $b_{i}$ are the learnable parameters of the network, and the sigmoid stimulation function is known as sigmoid.

The output of input gate $i_{t}$ is then used to compute a candidate's hidden state ${\tilde{h}}_{t}$ that includes the new input [equation (2)]:

(2) $${\tilde{h}}_{t}=\tanh\left(W\;\left[h_{t-1},i_{t}\times x_{t}\right]+b\right)$$

where, W and b are the learnable variables of the network; moreover, tanh is the hyperbolic tangent activation function.

### 5.2 Update gate

In a GRU, the gate of update ascertains what proportion of the prior concealed status ought to be kept, and what proportion of the incoming input ought be absorbed into the current concealed status. The gate of update receives the present input and the prior concealed status as outputs and inputs. A value ranging from 0 to 1 illustrates the maintenance fraction of the former hidden state. The number 0 indicates that no information from the previous hidden state is maintained, whereas the value 1 indicates that all information from the previous hidden state has been preserved. Mathematically, the update gate can be defined as follows [equation (3)]:

(3) $$z_{t}=sigmoid\left(W_{z}\left[h_{t-1},x_{t}\right]+b_{z}\right)$$

where, $h_{t-1}$ is the former concealed status, $x_{t}$ is the present input, $W_{z}$ and $b_{z}$ are the learnable parameters of the network, and sigmoid is the sigmoid function.

The output of the update gate $z_{t}$ is then used to compute the current hidden state $h_{t}$ as a weighted sum of the previous hidden state $h_{t-1}$ and the candidate hidden state ${\tilde{h}}_{t}$ [equation (4)]:

(4) $$h_{t}=z_{t}\times h_{t-1}+\left(1-z_{t}\right)\times {\tilde{h}}_{t}$$

The update gate allows the network to, only when necessary, update the concealed status regarding the input at each step, that can help avoid gradients disappearing or bursting over extended sequences.

### 5.3 Reset gate

In a GRU, the gate of reset ascertains what proportion of the prior concealed status ought to be forgotten. The reset gate accepts the present input and the former concealed status as outputs and inputs of a value ranging from 0 to 1 that illustrate the fraction of the former hidden state that should be forgotten. The number 0 indicates that all prior hidden states are forgotten, but the value 1 indicates that all previous hidden states are maintained. Mathematically, the reset gate can be defined as follows [equation (5)]:

(5) $$r_{t}=sigmoid\left(W_{r}\;\left[h_{t-1},x_{t}\right]+b_{r}\right)$$

The reset gate output $r_{t}$ is then used to compute the modified previous hidden state ${\tilde{h}}_{t}$ that includes the new input [equation (6)]:

(6) $${\tilde{h}}_{t-1}=r_{t}\times h_{t-1}$$

The modified former concealed status ${\tilde{h}}_{t-1}$ is then combined with the concealed status that is candidate ${\tilde{h}}_{t}$ using the update gate, as described above.

The gate of reset enables the network to, only when necessary, forget bits of the former concealed status that are not any more relevant to the present input, which can aid in preventing the training data from overfitting. In general, the GRU design enables the network to choose update gate and forget data at each step, which can aid in capturing long-term relationships in sequential data. The gating techniques also keep gradients from disappearing or bursting over extended sequences, making the network easier to be trained. It has been discovered that GRUs are effective in a number of sequential data processing applications and are frequently employed in the field of deep learning.

### 5.4 Objective function

The production values' mean square error is used as the valuation parameter for Gated Recurrent Unit (GRU) networks. When the weight configuration of the GRU networks is reduced, optimal network performance is attained. The Mean Squared Error is often represented by the symbol MSE. the metaheuristic approach suggested in this paper has been used to minimize this fitness function. The computational equation has been illustrated in the following [equation (7)]:

(7) $$MSE=\frac{1}{\tau_{pop}\;}\sum_{j=1}^{\tau }{\left(D_{ij}-Y_{ij}\right)}^{2}$$

where, τ specifies the size of test set, and $Y_{ij}$ and $D_{ij}$ represent the forecasted and desired amount of the GRU networks. The term pop defines the population size under investigation. In this study, a modified metaheuristic algorithm has been designed and utilized, called Northern Goshawk Optimization with NGO-LC to minimize the MSE function.

## 6 Northern Goshawk optimization with NGO-LC

### 6.1 Inspiration and behavior of northern goshawk

In the group of Accipitridae, there is a medium-large predator that is the northern goshawk. For the first time in 1758, Linnaeus in Systema Nature described it by the existing scientific title, i.e., Accipiter gentilis. One organ of the Accipiter species is the northern goshawk that chases a wide range of preys, including birds of tiny and big sizes and perhaps additional hunter birds, tiny mammals namely squirrels, rabbits, mice, and even animals like raccoons and foxes. The single organ of this species is the northern goshawk, which is spread in North America and Eurasia.

The female kind's length is from 58 to 69 cm and its weight is1220 gr, and also the space between the 2 wings is assessed to be between 108 and 127 cm. But for the male, the space between the two wings is between 89 and 105 cm, length of its bady varies from 46 to 61 cm, and it weighs approximately 780 gr.

The northern goshawk chasing approach contains two phases. In the first phase, after recognizing the prey, it flies toward it fast. In addition, in the second phase, it chases the hunt in a tiny tail hunting procedure. Manner of northern goshawk in chasing and grabbing prey is a smart procedure. The stated method's numerical modeling is the foremost motivation aimed at planning the suggested NGO process.

### 6.2 Algorithm initialization process

The suggested northern goshawk optimization is on the basis of the population procedure that northern goshawks are these algorithm's hunter organs. In Northern Goshawk Optimization Algorithm, every individual identifies a suggested result to the trouble that specifies the volume of element. Every population individual is a carrier, sometimes called vector, and these carriers altogether organize a community of an algorithm in a matrix. In the algorithm's commencement, the individuals have been initialized in the solution space randomly. The following equation determines the population matrix of the suggested NGO algorithm [equation (8)].

(8) $$Z={\left[\begin{array}{c} Z_{1}\\ \vdots \\ Z_{i}\\ \vdots \\ Z_{N} \end{array}\right]}_{N\times m}=\left[\begin{array}{c} \begin{array}{cc} z_{1,1} & \ldots \end{array}\;\begin{array}{ccc} z_{1,d} & \ldots & z_{1,m} \end{array}\\ \begin{array}{cc} \vdots & \ddots \end{array}\;\begin{array}{ccc} \vdots & \ddots & \vdots \end{array}\\ \begin{array}{cc} z_{i,1} & \ldots \end{array}\;\begin{array}{ccc} z_{i,d} & \ldots & z_{i,m} \end{array}\\ \begin{array}{cc} \vdots & \ddots \end{array}\;\begin{array}{ccc} \vdots & \ddots & \vdots \end{array}\\ \begin{array}{cc} z_{N,1} & \ldots \end{array}\;\begin{array}{ccc} z_{N,d} & \ldots & z_{N,m} \end{array} \end{array}\right]$$

where, the northern goshawks' population is defined by Z, $Z_{i}$ is the $i^{th}$ suggested result, and $z_{i,j}$ is the $j^{th}$ variable amount that is determined by the $i^{th}$ suggested result. The population individuals and the number of problem variables are defined by N and m, respectively. As mentioned before, solutions to the problems can be gained by the individuals in the community. Consequently, the problem ‘s cost mechanism could be assessed on the basis of every population individual. The amount of these cost functions is determined as a vector by the next formula [equation (9)].

(9) $$P\left(Z\right)={\left[\begin{array}{c} P_{1}={P(Z}_{1})\\ \vdots \\ {P_{i}=P(Z}_{i})\\ \vdots \\ {P_{N}=P(Z}_{N}) \end{array}\right]}_{N\times 1}$$

where, the amount of calculated cost function vector is specified by P, and $P_{i}$ is the objective cost amount calculated by the $i^{th}$ suggested result. The standard for determining the greatest result is the cost function's amount. While solving maximization issues, the massive amount of cost mechanism should be solved. While solving minimization issues, the little amount of cost mechanism should be solved; consequently, the better suggested result is gained. With respect to every iteration, new amounts are obtained for the cost function; the best-suggested result must be renewed in every iteration.

### 6.3 Mathematical modeling of proposed NGO

In the plan of the suggested NGO process to renew the population of individuals, the northern goshawk approach simulation while preying has been utilized. This approach is designed based on the two important manners of northern goshawk that are:

− Hunt recognition and assault.
− The pursuit and runaway processes are simulated in two stages.

**Fig. 5:**
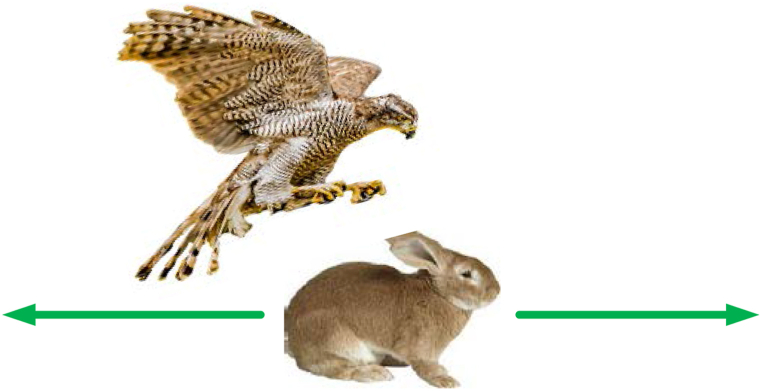
Scheme of quarry choice and assault by a northern goshawk.

Fig. (5) shows the northern goshawk's approach and manner in chasing time in nature.

Stage 1 Prey Identification (Exploration)

**Fig. 6:**
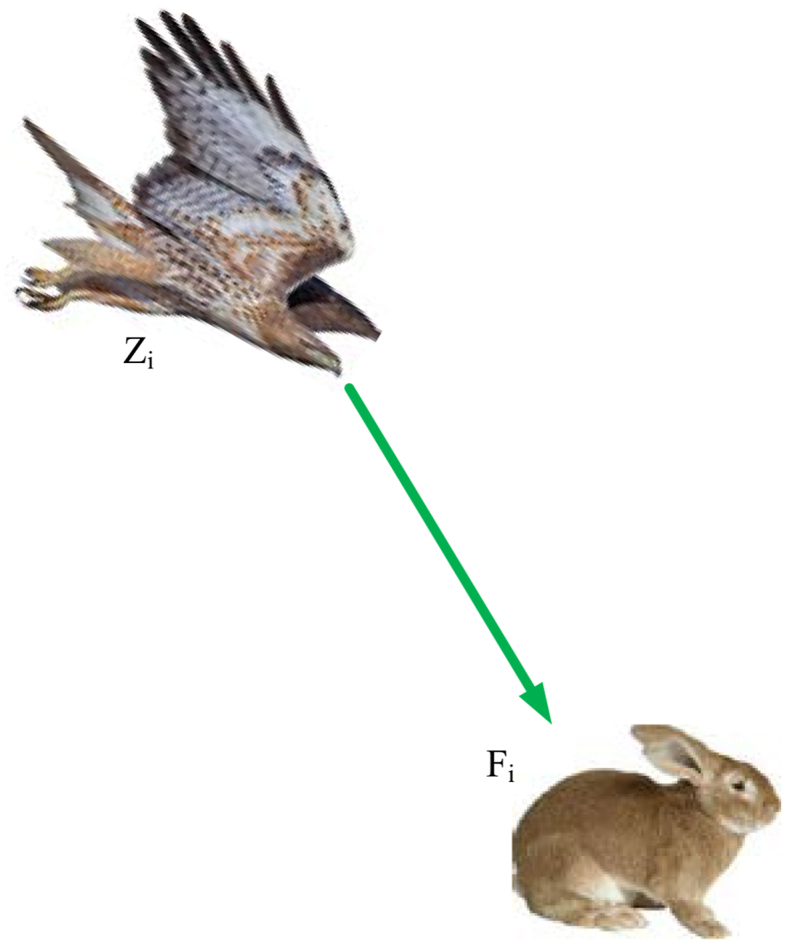
Diagram of the pursuit between northern goshawk and prey.

In first stage of preying, the Northern goshawk chooses quarry randomly and then rapidly assaults it. This stage boosts the Northern goshawk optimization's assessment power in light of the quarry's choice in the solution space, which is done randomly. The current step makes an exploration zone be a global search that the goal is recognizing the ideal zone. An illustration of the northern goshawk's behavior at this stage of choosing and attacking the prey is demonstrated in Fig. (6).

The first stage's theories are mathematically determined by the next formulas [equations (10), (11), (12)].

(10) $$F_{i}=Z_{k\;}\;i=1,2,\ldots ,N\;k=1,2,\ldots ,i-1,i+1,\ldots ,N$$

(11) $$z_{i,j}^{new,f1}=\left\{ \begin{array}{c} z_{i,j}+r\left(f_{i,j}-Iz_{i,j}\right),P_{F_{i}}<\;P_{i}\\ z_{i,j}+r\left(z_{i,j}-f_{i,j}\right),P_{F_{i}}\geq P_{i} \end{array}\right.$$

(12) $$Z_{i}=\left\{ \begin{array}{c} Z_{i}^{new,f1}\;P_{i}^{new,f1}<\;P_{i}\\ Z_{i}\;P_{i}^{new,f1}\geq P_{i} \end{array}\right.$$

where, the quarry's situation for the $i^{th}$ northern goshawk is defined by $F_{i}$, $P_{i}$ is cost mechanism amount, k is a number, which is natural and chosen at random, that is ranged between [1, N], $Z_{i}^{new,f1}$ is the novel state of the $i^{th}$ suggested result, $z_{i,j}^{new,f1}$ is its $j^{th}$ dimension, $P_{i}^{new,f1}$ is its cost function amount on the basis of first stage of Northern Goshawk Optimization, r is a number, chosen at random, that is from 0 to 1; moreover, I is a number, chosen at random, that is one or two.

Stage 2 Exploitation Stage

Having attacked the hunt by the northern goshawk, the quarry strives to run away. Consequently, in a pursue and hunt procedure, NG (Northern Goshawk) keeps on following the hunt. Because of the northern goshawks' high speed, they could follow their hunt in every condition and finally catch their prey. The imitation of manner boosts the process's utilization power in a solution space's local search. In the suggested NGO process, it is supposed that this preying is near the assault location within a radius of R. Fig. 4 illustrates the pursuit procedure between the northern goshawk and the prey. The second stage's descriptions are mathematically determined by the following equations [equations (13), (14), (15)].

(13) $$R=\frac{2}{100}\left(1-\frac{t}{T}\right)$$

(14) $$z_{i,j}^{new,f2}=z_{i,j}+R\left(2r-1\right)z_{i,j}$$

(15) $$Z_{i}=\left\{ \begin{array}{c} Z_{i}^{new,f2}\;P_{i}^{new,f2}<\;P_{i}\\ Z_{i}\;P_{i}^{new,f2}\geq P_{i} \end{array}\right.$$

where, t is the quantity of iterations, T is the highest number of iterations, $Z_{i}^{new,f2}$ is the new conditions for $i^{th}$ suggested result, $z_{i,j}^{new,f2}$ is its $j^{th}$ dimension, $P_{i}^{new,f2}$ is its cost function amount that is on the basis of NGO's second stage.

3) Flowchart of Northern Goshawk Optimizer, iterative process, and pseudo-code

Since then, all population's individuals have been renewed on the basis of the first and second stages of the suggested NGO process, an iterative process is done. Moreover, the new amounts of the population individuals, the cost function, and the greatest suggested result are specified. Afterward, the process passes in the following iteration and the renewal of the population's individuals keeps on the basis of Eq. (10) to Eq. (15) until it reaches the final iterative process. Finally, after the complete operation of the NGO, the greatest suggested result obtained throughout the iterative process is presented as a quasi-optimal result aimed at a certain optimization problem.

### 6.4 Northern Goshawk Optimizer with Lévy flight and chaos theory (NGO-LC)

The NGO algorithm is a metaheuristic optimization algorithm that has demonstrated promising findings to solve some of those problems. However, like all refining algorithms, it has some limitations and can become trapped in local optima, which can lead to suboptimal solutions. Therefore, there is a need to modify the NGO algorithm to improve its performance and make it more robust. The modified version of the algorithm, which is called Northern Goshawk Optimization with Lévy flight and chaos theory (NGO-LC), incorporates two modifications, namely the use of chaos theory and Lévy flight.

L (Lévy Flight) is a kind of walk, done at random, where the step lengths follow a distribution of probabilities known as the Lévy distribution. By incorporating L flight into the NGO algorithm, the search process becomes more exploratory, enabling the algorithm to leave local optima and discover superior solutions. This is because Lévy flight has a higher probability of taking larger steps, which can help the algorithm to quickly search a larger space and find global optima. The Lévy flight ($F_{l}\left(\alpha \right)$) relies on random walk theory that can be achieved as follows [equations (16), (17), (18)]:

(16) $$F_{l}\left(\alpha \right)\approx \frac{1}{\beta^{1+\tau }}$$

(17) $$\alpha =\frac{A}{{\left|B\right|}^{\frac{1}{\tau }}},A,B∼N\left(0,\sigma^{2}\right)$$

(18) $$\sigma^{2}={\left\{\frac{\sin\;\left(\pi \tau /2\right)}{2^{\left(1+\tau \right)/2}}\times \frac{\Gamma \left(1+\tau \right)}{\tau \Gamma \left(\left(1+\tau \right)/2\right)}\right\}}^{\frac{2}{\tau }}$$

where, β, Γ(.), and τ represent the step size, the Gamma function, and the Lévy flight constant, respectively. In this study, τ=1.5 [21]. By applying this mechanism to the Stage one, the following formula can be achieved [equation (19)]:

(19) $$z_{i,j}^{new,f1}=\left\{ \begin{array}{c} z_{i,j}+F_{l}\left(\alpha \right)\times \left(f_{i,j}-Iz_{i,j}\right),P_{F_{i}}<\;P_{i}\\ z_{i,j}+F_{l}\left(\alpha \right)\times \left(z_{i,j}-f_{i,j}\right),P_{F_{i}}\geq P_{i} \end{array}\right.$$

The actions of dynamic systems that are extremely sensitive to the starting point of the situations are the subject of the mathematical concept known as Chaos theory. By incorporating chaos theory into the NGO-LC algorithm, the search process becomes more chaotic, which can help to prevent the algorithm from becoming stuck in local optima. This is because chaos theory allows for the introduction of randomness into the search process, which is able to assist the algorithm to discover new areas to search and discover superior solutions. The chaos theory could be mathematically described as below [equation (20)]:

(20) $$\begin{array}{c} {CM}_{i+1}^{j}=f\left({CM}_{i}^{j}\right)\\ j=1,2,\ldots ,M \end{array}$$

where, $f\left({CM}_{i}^{j}\right)$ stands for the generator function [22].

By applying the chaos function to the second stage's descriptions, it can be reformulated as follows [equations (21), (22)]:

(21) $$z_{i,j}^{new,f2}=z_{i,j}+\gamma_{i}\times \left(2r-1\right)z_{i,j}$$

where,

(22) $$\gamma_{i+1}={\left(\gamma_{i}\right)}^{2}\;\sin\left(\pi \gamma_{i}\right)$$

where, $\gamma_{0}=rand\left(.\right)$.

Incorporating Lévy flight and chaos theory into the NGO-LC algorithm offers several advantages. Firstly, Lévy flight enables the algorithm to efficiently search a larger space and identify global optima, ultimately enhancing its performance. Secondly, chaos theory helps algorithm not to be trapped in current condition, which can be called local optima, that results in superior solutions. Lastly, the combination of Lévy flight and chaos theory provides an improved search process, allowing the algorithm to discover more amazing solutions in a shorter period of time. Generally, modifying the NGO algorithm with Lévy flight and chaos theory makes it more robust and efficient, enabling it to solve complex optimization problems in fields such as engineering, finance, and biology.

### 6.5 Algorithm authentication

The modified metaheuristic, Northern Goshawk Optimization with Lévy flight and chaos theory (NGO-LC), was evaluated using a desktop computer with 16 GB of RAM and Intel Core i7 processor (3.4 GHz). The algorithm was implemented in Matlab R2017b programming language. To assess the efficiency of the NGO-LC algorithm, it was examined on standard benchmark functions, including the Powell Sum, Quartic, Zakharov, Squares, Rosenbrock's Su, Step, Schwefel 2.23, Schwefel 2.22, Schwefel 2.21, Schwefel 2.20, Schwefel 1.2, and Sphere functions. The findings were contrasted with five modern optimization strategies, comprising Tunicate Swarm Algorithm (TSA) [23], GSA (Gravitational Search Algorithm) [24], MVO (Multi-Verse Optimizer) [25], Pigeon-Inspired Optimization (PIO) algorithm [26], and the original NGO (Northern Goshawk Optimization) [27]. To ensure the reliability of the results, all algorithms were run 20 times on all test functions. The average values of the objective function, were recorded and compared among the different algorithms.

Table 2 provides information regarding the benchmark functions used to validate the algorithms. It is noteworthy that the minimum value of all functions is 0.

**Table 2:** Information regarding the benchmark functions used to validate the algorithms.

Test Function	Formulation	Dimension	Range
Sphere	$F_{1}\left(x\right)=\sum_{i=1}^{d}x_{i}^{2}$	30	[−100, 100]
Schwefel 1.2	$F_{2}\left(x\right)=\sum_{i=1}^{d}{\left(\sum_{j=1}^{i}x_{j}^{2}\right)}^{2}$	30	[−100, 100]
Schwefel 2.20	$F_{3}\left(x\right)=\sum_{i=1}^{d}{|x}_{i}|$	30	[−100, 100]
Schwefel 2.21	$F_{4}\left(x\right)=\max_{i=1,2,\ldots ,d}{|x}_{i}|$	30	[−100, 100]
Schwefel 2.22	$F_{5}\left(x\right)=\sum_{i=1}^{n}{|X}_{i}|+\prod_{i=1}^{n}\left|x_{i}\right|$	30	[−10, 10]
Schwefel 2.23	$F_{6}\left(x\right)=\sum_{i=1}^{d}x_{i}^{10}$	30	[−10, 10]
Rosenbrock's	$F_{8}\left(x\right)=\sum_{i=1}^{n-1}\left[100{\left(x_{i+1}-x_{i}^{2}\right)}^{2}+{\left(x_{i}-1\right)}^{2}\right]$	30	[-30,30]
Step	$F_{7}\left(x\right)=\sum_{i=1}^{d}{\left(x_{i}+0.5\right)}^{2}$	30	[−100, 100]
Sum Squares	$F_{9}\left(x\right)=\sum_{i=1}^{d}ix_{i}^{2}$	30	[−10, 10]
Zakharov	$F_{10}\left(x\right)=\sum_{i=1}^{d}x_{i}^{2}+\sum_{i=1}^{d}{\left(0.5ix_{i}\right)}^{2}+\sum_{i=1}^{d}{\left(0.5ix_{i}\right)}^{4}$	30	[-5,10]
Quartic	$F_{11}\left(x\right)=\sum_{i=1}^{d}x_{i}^{4}+rand\left(0,1\right)$	30	[−1.28, 1.28]
Powell sum	$F_{12}\left(x\right)=\sum_{i=1}^{d}{|x}_{i}{|}^{i+1}$	30	[−1, 1]

It's critical to contrast the outcomes of the Northern Goshawk Optimizer with Lévy Flight and Chaos Theory (NGO-LC) with those of five other cutting-edge optimization techniques to assess the efficiency of the procedure. These techniques are used as the basis for assessing the efficiency and competitiveness of the suggested algorithm since they are extensively acknowledged and approved in the area. Table 3 displays the parameter values used in the studied algorithms.

**Table 3:** Parameter values of the investigated algorithms.

Algorithm	Parameter	Value
TSA (Tunicate Swarm Algorithm) [23]	Search agents	100
	$P_{\min}$	2
	$P_{\max}$	3
	Quantity of generations	900
Gravitational Search Algorithm (GSA) [24]	Agents for Search	100
	A constant in gravity	80
	Coefficient of alpha	50
	Quantity of generations	900
Multi-Verse Optimizer (MVO) [25]	$WEP_{\min}$	0.1
	$WEP_{\max}$	2
	Coefficient(P)	8
Pigeon-Inspired Optimization (PIO) algorithm [26]	Number of Pigeons	500
	Dimension of space	15
	Compass factor and map	0.5
	Compass operation and map limit	200
	Operation limit of landmark	150
	Inertia factor (w)	2
	Self-confidence factor ($c_{1}$)	1.5
	Swarm confidence factor ($c_{2}$)	1.5

The Northern Goshawk Optimization Method with Lévy Flight and Chaos Theory and the five cutting-edge algorithms are run 20 times on each benchmark function to guarantee accurate and statistically meaningful results. Several runs of the algorithms assist reduce the impact of random fluctuations and offer a more thorough assessment of their efficacy. Based on the following criteria, the NGO-LC and cutting-edge approaches are compared in terms of performance, which is the most accurate measurement of how closely discovered solutions resemble best-known or ideal solutions.

Average accuracy: Calculate the average accuracy after 20 trials.

StD: The accuracy of standard deviation value.

To identify the strengths and limitations of the recommended algorithm, the execution findings are contrasted and analyzed. Table 4 displays comparison findings of the studied optimization algorithms.

**Table 4:** Comparison findings of the studied optimization algorithms.

	TSA [23]	GSA [24]	MVO [25]	PIO [26]	NGO [27]	NGO-LC
Best	3.44	249.39	0.00	0.00	164.35	0.00
Min	10.23	532.73	0.00	0.00	496.55	0.00
StD	4.03	447.40	0.00	0.00	251.57	0.00
Best	498.32	2972.40	43095.00	10.48	816.31	0.25
Min	1415.10	7040.50	59362.00	98.42	3533.70	1.21
StD	480.15	3506.90	24973.00	108.18	2160.00	1.01
Best	3.47	54.01	0.00	0.04	63.35	0.00
Min	10.33	83.33	0.00	0.07	129.95	0.00
StD	2.33	18.30	0.00	0.02	20.04	0.00
Best	3.40	12.14	0.08	0.35	6.24	0.00
Min	5.94	26.17	38.01	0.52	8.97	0.00
StD	1.28	5.12	30.59	0.28	2.43	0.00
Best	0.32	6.48	0.00	0.00	6.98	0.00
Min	0.93	7.79	0.00	0.01	11.37	0.00
StD	0.32	2.83	0.00	0.00	3.03	0.00
Best	0.00	0.16	0.00	0.00	0.27	0.00
Min	0.00	963.78	0.00	0.00	183.06	0.00
StD	0.01	2548.80	0.00	0.00	331.04	0.00
Best	3.78	139.78	0.42	1.33	158.35	0.04
Min	12.15	868.44	1.21	2.03	570.77	0.18
StD	4.49	498.05	0.41	0.57	197.68	0.09
Best	154.82	7367.50	15.41	16.08	5860.10	24.05
Min	544.07	98370.00	17.14	29.79	52256.00	15.46
StD	279.92	67621.00	0.05	14.40	63835.00	0.27
Best	0.46	44.48	0.00	0.00	17.56	0.00
Min	2.08	110.95	0.00	0.00	74.18	0.00
StD	0.64	145.19	0.00	0.00	23.30	0.00
Best	32.67	53.99	154.60	0.53	147.20	5.69
Min	69.08	113.33	392.72	10.87	361.86	19.29
StD	44.76	45.22	69.58	10.37	51.92	14.77
Best	0.02	0.04	0.00	0.00	0.14	0.00
Min	0.06	0.14	0.02	0.01	0.24	0.01
StD	0.02	0.07	0.02	0.00	0.13	0.00
Best	0.00	0.00	0.00	0.00	0.00	0.00
Min	0.00	0.00	0.00	0.00	0.00	0.00
StD	0.00	0.00	0.00	0.00	0.00	0.00

The findings demonstrate that the NGO-LC algorithm outperformed all the algorithms regarding the best objective function value. It achieved the best value of 0.00 on five out of nine benchmark functions, while the other algorithms achieved the best value of 0.00 on only one or two functions. This indicates that the NGO-LC algorithm is highly effective in finding the global optima on these functions. In addition, the NGO-LC algorithm achieved the lowest values for standard deviation on most of the benchmark functions, which indicates that it is more stable and consistent in its performance across different runs.

The comparison results demonstrate that NGO-LC theory algorithm is the best one among the studied optimization methods for solving the benchmark functions. The use of LC theory in the NGO-LC algorithm provides several advantages, including efficient search of a larger space, prevention of being trapped in current condition which is called local optima, and improved search process. Therefore, the NGO-LC algorithm has the potential to be applied in many fields that require optimization.

## 7 Results and discussions

In the following part, the results of the simulations were investigated, which were run utilizing the suggested medical image diagnosis methodologies to identify the oral cancer. The simulation findings are assessed and presented in a clear path, as they are the methodologies utilized to achieve these results. Furthermore, a detailed explanation of the relevance and significance of these findings has been given in the context of the research. The research focuses on the potential influence of these approaches on timely identification and diagnosis of mouth cancer; moreover, they are advantageous for patients and healthcare professionals.

### 7.1 Experimental setup

The investigations were carried out using a dataset of oral cancer images, with a focus on the lips and tongue areas. The collection included 131 images, with 87 images classified as cancerous and 44 images classified as non-cancerous. For the classification task, a deep learning-based algorithm has been used employing a Gated Recurrent Unit (GRU) network architecture. The simulations in this paper were carried out utilizing a specific hardware setup and software environment.

The simulations were run on a robot with an Intel® Pentium® processor CPU G645 which runs at 2.90 GHz and 2 GB of RAM. The accessibility of resources and the practicality of executing the simulations within the stated limitations influenced the selection of this hardware setup. The coding environment for the construction of the picture preprocessing methods and the deep learning algorithm was MATLAB R2017b. MATLAB has a broad range of tools and libraries for image processing, deep learning, and data analysis, making it well-suited to the tasks involved in the present study.

The simulations were executed by running MATLAB programs that included the necessary image preprocessing stages, GRU network training, and a classification stage. The machine configuration's processing power and memory capacity were adequate to meet the computational needs of the simulations.

### 7.2 Simulation results

The simulations were conducted using a 70/30 train-test split on the preprocessed dataset. The GRU network was trained on the set of training, and its performance was assessed on the test set. The optimized network has been designed by NGO-LC, which is called GRU/NGO-LC. Several evaluation metrics were used, including precision, accuracy, specificity, sensitivity, F1-score, and MCC metrics which are defined in the following [equations (23), (24), (25), (26), (27), (28)]:

(23) $$MCC=\frac{TP\times TN-TP\times FN}{\sqrt{\left(TP+FP\right)\times \left(TP+FN\right)\times \left(TN+FP\right)\times \left(TN+FN\right)}}\times 100$$

(24) $$Fs=2\times \frac{Precision\times Sensitivity}{Precision+Sensitivity}\times 100$$

(25) $$Accuracy=\frac{TP+TN}{TP+TN+FP+FN}\times 100$$

(26) $$Sensitivity=\frac{TP}{TP+FN}\times 100$$

(27) $$Precision=\frac{TP}{TP+FP}\times 100$$

(28) $$Specificity=\frac{TN}{TN+FP}\times 100$$

To classify the model's output, the labels False Positive (FP), False Negative (FN), True Positive (TP), and True Negative (TN) are utilized. A TP outcome happens when the system predicts a positive event properly. A True Negative result happens when the model accurately predicts a bad outcome. An FP outcome occurs when the system predicts a good result that is really negative. A False Negative outcome, on the other hand, happens when the system forecasts a bad result that is, however, really positive. Using these terminologies, it can result in a better understanding of the model's accuracy and efficacy for categorization purposes.

First, a performance study of the suggested technique has been given with and without preprocessing. In this case, the data trained the GRU network on the original, unbalanced dataset, and after image preprocessing, then the outcomes were compared. This experiment's primary findings are presented in the publication, including how utilizing oversampled data might enhance sensitivity and F1-score. Additionally, it talks about some of the methods' drawbacks, such the potential bias brought on by oversampling. Table 5 displays the outcomes of the oral cancer, including Lips and Tongue image dataset identification both before and after preprocessing.where, NA means that the result is not applicable because of a zero division. The findings of Table 4 show that preprocessing offers the potential to improve the effectiveness of the oral cancer identification method for the oral cancer image dataset. As a result, the preprocessing and prediction models' architecture, which has been suggested, perhaps could significantly refine the precision and reliability of oral image analysis, which has crucial medical consequences for identifying and treating knee injuries and diseases. Therefore, it can be conceded that the quality of the oral cancer image dataset can be improved by implementing the previously suggested preprocessing procedures.

**Table 5:** The outcomes of diagnosing the mouth cancer on the oral cancer image dataset before and after the application of preprocessing.

Method	Precision	Accuracy	F1-score	Specificity	MCC	Sensitivity
Before preprocessing	96.14	95.11	NA	97.05	86.92	NA
After preprocessing	98.54	98.34	98.55	98.67	87.38	98.60

The performance of a given approach for optimizing the GRU networks should be compared to that of other optimization strategies in the next step in order to show its efficacy. In particular, a comparison between the suggested approach and two currently used methods, including the normal Gated Recurrent Network and a Gated Recurrent Network optimized by the traditional NGO optimization, should be made.

The goal of this comparison is to see if the suggested technique surpasses the two current methods in various ways. The previously mentioned metrics were used once more to make this comparison fair and accurate. This will help guarantee that any disparities in performance are not merely attributable to chance or bias imposed by specific experimental settings.

The oral cancer picture collection was used to address simulations, which were validated by comparing the two aforementioned methodologies.

The results of the oral cancer recognition based on GRU/NGO-LC, GRU/NGO, and the standard GRU on the oral cancer image database have been illustrated in Table 6.

**Table 6:** Findings of the oral cancer recognition based on GRU/NGO-LC, GRU/NGO, and the standard GRU on the oral cancer image data source.

Methodology	Precision	Accuracy	Specificity	Sensitivity	F1-score	MCC
GRU/NGO-LC	98.54	98.34	98.67	98.60	98.55	87.38
GRU/NGO	83.68	91.27	95.75	93.80	93.27	78.32
GRU	79.64	90.54	93.86	90.58	89.63	60.32

Three different methods of diagnosing oral cancer are depicted in Table 5: GRU/NGO-LC, GRU/NGO, and the standard GRU, all tested on the Oral Cancer image dataset. The findings display that the recommended GRU/NGO-LC strategy gained the highest MCC, F1-score, precision, specificity, sensitivity, and accuracy, with values of 87.38 %, 98.55 %, 98.54 %, 98.67 %, 98.60 %, and 98.34 %, respectively. The GRU/NGO method achieved a lower accuracy of 91.27 % but still performed well regarding sensitivity, specificity, precision, F1-score, and MCC, with values of 93.80 %, 95.75 %, 83.68 %, 93.27 %, and 78.32 %, respectively. The standard GRU method achieved an accuracy of 90.54 % with lower scores in all other metrics, for instance specificity which was the lowest among the three methods at 93.86 %. Overall, the results suggest that the proposed methods, particularly GRU/NGO-LC, outperform the standard GRU method for oral cancer recognition. These results are of paramount significance for the development of effective and accurate medical image diagnosis techniques for oral cancer, which can have significant benefits for patients and healthcare providers.

To certify the suggested strategy, its results are compared with other recently published networks, such as Lightweight Deep Convolutional Neural Network (LDNN) [28], ResNet-101 [29], Faster R–CNN [29], optimized Probabilistic Genetic Algorithm Neural Network (NNPGA) models [30], and Transfer learning (TFL) neural network [31]. This analysis occurred to assess the efficiency of the suggested strategy in improving oral cancer detection outcomes. Table 7 indicates the results of the mouth cancer recognition dependent on the studied methods on the oral cancer image dataset.

**Table 7:** Findings of the oral cancer recognition dependent on the studied methods on the oral cancer image data source.

Method	Accuracy	Precision	Specificity	F1-score	Sensitivity
GRU/NGO-LC	98.34	98.54	98.67	98.55	98.60
LDNN [28]	82.47	94.01	94.59	87.94	84.80
ResNet-101 [29]	91.95	93.02	76.21	83.22	86.71
R–CNN [29]	90.51	91.35	82.68	85.88	82.48
NNPGA [30]	81.09	69.59	82.74	98.36	96.80
TFL [31]	90.36	84.78	90.89	82.01	97.00

It can be seen from the results in Table 7 that the suggested strategy, GRU/NGO-LC, achieved an accuracy of 98.34 %, precision of 98.54 %, specificity of 98.67 %, F1-score of 98.55 % and sensitivity of 98.60 %. Compared to other methods, such as LDNN, ResNet-101, R–CNN, and NNPGA, GRU/NGO-LC outperformed them in terms of accuracy and precision by a significant margin while also achieving high values for specificity and sensitivity, which are critical metrics in medical diagnosis. Transfer Learning (TFL) neural network performed well with an accuracy score close to that obtained by GRU/NGO-LC but had lower scores for other metrics like precision and F1-score compared to GRU/NGO-LC.

**Fig. 7:**
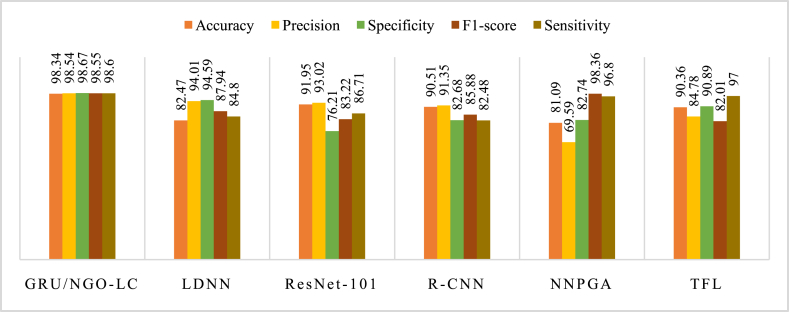
Bar plot representation of the oral cancer recognition based on the studied methods on the oral cancer image dataset.

The bar plot of Table 6 is shown in Fig. (7). This plot provides a visual representation of the performance of the proposed GRU/NGO-LC model relative to other modern designs for oral cancer recognition. The plot clearly demonstrates the better performance of the suggested algorithm over other current modern models, effectively showcasing its effectiveness and competitiveness in the field. The figure serves as a valuable contribution to the paper, providing a concise and easily interpretable summary of the results and highlighting the potential effect of the suggested strategy on the development of clinical image diagnosis techniques for oral cancer recognition.

Consequently, the findings of the simulations indicate that careful selection and application of image preprocessing techniques are crucial for optimizing the efficiency of advanced learning-based algorithms in oral cancer detection tasks. The improvements in classification accuracy and other evaluation metrics validate the potential of these techniques as valuable tools for fast screening, earlier detection, and therapeutic efficacy assessment of oral cancer.

## 8 Limitations and future directions

In this study, an investigation was conducted in our work that focused on a new method for diagnosing oral cancer. This involved utilizing Gated Recurrent Unit (GRU) networks that were optimized using an enhanced version of the Northern Goshawk Optimization (NGO) algorithm. This approach offers numerous benefits, including the ability to analyze extensive and complex datasets, achieve high levels of accuracy, and enable the early detection of oral cancer. The enhanced NGO algorithm plays a vital role in enhancing the performance of GRU networks and improving diagnostic accuracy. What sets this research apart from other AI studies in this field is its unique emphasis on optimizing GRU networks with the NGO algorithm, which not only enhances accuracy but also enables early detection of oral cancer. While previous studies have explored various techniques such as Convolutional Neural Networks (CNNs), capsule networks, and mobile-based classification methods, the innovative approach combines GRU networks with a state-of-the-art optimization strategy. The potential impact of the present work lies in its ability to advance early diagnosis and improve prognosis prediction of individuals affected by oral cancer.

The study's limitations encompass potential bias and generalization issues stemming from the training data, the lack of interpretability in deep learning models, and the necessity for clinical validation in real-world scenarios. Overcoming these constraints will entail endeavors to improve interpretability of the model, partnerships with clinicians and pathologists, and adherence to ethical and regulatory standards. Future research directions involve merging histopathological images with other modalities, assessing the model's performance longitudinally, delving into transfer learning from related medical fields, designing user-friendly clinical decision support systems, and crafting patient-centric applications for early self-assessment and risk estimation. Collaboration with healthcare institutions and involvement in interdisciplinary research are also vital to meet clinical requirements and ensure the widespread acceptance of AI technology in oral cancer diagnosis.

## 9 Conclusions

Early diagnosis of oral cancer is extremely vital to successfully cure the patients and improve consequences. When an oral cancer is detected and treated in its early stages, the chances of survival and recovery are significantly higher. Timely detection of oral cancer involves regular dental check-ups and oral cancer screenings. During these screenings, a dentist or healthcare professional will examine the mouth and throat for any signs of abnormal growths or lesions. They may also perform a biopsy to confirm the presence of cancerous cells. Early diagnosis of oral cancer is critical to improve consequences and reduce the impact of the disease. Regular dental check-ups, awareness of symptoms, and risk factor reduction can all play important roles in achieving early diagnosis and successful treatment of oral cancer. Medical imaging has a key role in the timely detection of oral cancer. Some of the techniques of imaging, such as CT, MRI, and PET, can assist to detect oral cancer in its prior period.

clinical techniques of imaging, such as computed tomography, magnetic resonance imaging, and positron emission tomography scans, can aid to diagnosis oral cancer beforehand by detecting abnormal growths or tumors in the oral cavity. These imaging techniques can provide valuable information to healthcare professionals, allowing for prompt diagnosis and treatment. Regular dental check-ups and oral cancer screenings that have been combined with awareness of symptoms are also important for early detection and improved outcomes. This research paper presented a novel approach for the diagnosis of oral cancer using GRU networks optimized by a refined model of the NGO algorithm. The proposed GRU/NGO-LC demonstrated high accuracy and was capable of improving the early identification and treatment of oral cancer. The study made an important addition to medical science field, and the proposed method could be further developed and applied to other types of cancer and diseases. The results of the study also demonstrated the capability of using artificial intelligence algorithms and optimization techniques to improve the accuracy and efficiency of medical diagnosis and treatment. Generally, this research paper presented a significant step in establishing beneficial, dependable, and reliable techniques to the detection and diagnosis of oral cancer. Ultimately, the results demonstrated that the proposed approach achieved both high accuracy and specificity in diagnosing oral cancer, outperforming other modern strategies. The results of the research suggested the recommended approach had the potential to strengthen the meticulous and effective ways to diagnose oral cancer. Further investigations can discover the clinical implementation of the recommended technique and validate its effectiveness.

## Data availability statement

Research data are not shared.

## CRediT authorship contribution statement

**Lei Zhang:** Formal analysis, Data curation, Conceptualization. **Rongji Shi:** Formal analysis, Data curation, Conceptualization. **Naser Youssefi:** Formal analysis, Data curation, Conceptualization.

## Declaration of competing interest

The authors declare that they have no known competing financial interests or personal relationships that could have appeared to influence the work reported in this paper.
